# Assessing EFL (English as Foreign Language) Education for Sustainable Development: Exploring the Cultural Teaching Literature

**DOI:** 10.3390/ejihpe14080152

**Published:** 2024-08-07

**Authors:** Shujie Wu, Zahid Shafait

**Affiliations:** 1 Institute of Higher Education, Jinling Institute of Technology, Nanjing 211169, China; wushujie@zjnu.edu.cn; 2 College of Education, Zhejiang Normal University, Jinhua 321004, China

**Keywords:** cultural teaching, EFL, bibliometric analysis, interculturality, education for sustainable development

## Abstract

Cultural teaching is the underlying core component of English as Foreign Language (EFL) education. Although the previous literature has intensely studied this theme, a comprehensive bibliometric analysis of research characteristics and trends in this field is still lacking regarding cultural teaching in EFL education. This study aims to explore the research distribution, research hot topics, and research the trends of EFL cultural teaching by conducting a bibliometric analysis of 358 articles on Web of Science using CiteSpace. The analysis leads to the following three findings: (a) The countries that teach EFL prevail in terms of number of publications but lag behind in terms of research influence, and a global academic community has not taken shape. (b) Interculturality is the central theme, encompassing a range of related topics such as intercultural competence, intercultural communication, and cultural awareness, with key teachers, learners, and textbooks as research perspectives. (c) Multilingual turn has emerged as a prominent new trend, which emphasizes the importance of cultural diversity and pays more attention to source/native languages and cultures. Furthermore, possible measures of promoting interculturality were discussed on the basis of relevant literature studies. In addition, scholars are suggested to pay more academic attention to the research and practices of EFL countries.

## 1. Introduction

Education for Sustainable Development (ESD) was acknowledged by UNESCO as an essential component of achieving the Sustainable Development Goals (SDGs), and language is one of the core subjects that must integrate with the ESD [1]. Moreover, enhanced communication is a fundamental part of sustainable development [2]. English as a global language has become one of the most important instruments for worldwide communication, so English language education programs are actively advocating for sustainable foreign language teaching and learning [3]. Therefore, sustainable English as Foreign Language (EFL) education is of great importance for EFL students and learners in terms of sustainable development. Language is a medium of communication as a sociological trend that gives a significant aspect of culture and a reflection that depicts a country’s customs and community [4]. Since language and culture are interrelated, interdependent, inter-reliant and always move together in any form of communication [5], EFL education involves the underlying cultural aspects. Culture teaching refers to integration of culture into the language teaching programs and has an effect on motivation of the language learners and the process of teaching and learning [6]. Therefore, cultural teaching is thus an indispensable topic to sustainable EFL education for enhanced communication.

EFL education involves the culture of English language and the cultures of native languages, so it is essentially an intercultural education. UNESCO issued guidelines on intercultural education in 2006, which define in detail the role, objectives, and principles of intercultural education. Multiculturalism and interculturalism were regarded as the foundations of intercultural education, whose objective was therefore defined as to go beyond passive coexistence, to achieve a developing and sustainable way of living together in multicultural societies through the creation of understanding, respect, and dialogue between the different cultural groups [7]. For example, in China, cultural awareness was listed as one of the five teaching objectives in the English Curriculum Standards for Senior High School (experimental draft) issued in 2003 [8]. According to the curriculum standards, cultural awareness consists of four aspects: knowledge about culture, understanding of culture, cultural awareness and intercultural communicative competence. The College English Curriculum Requirements enacted by the Chinese Ministry of Education clearly state that College English is an educational system that is guided by foreign language teaching theories, regards English language knowledge and application skills, intercultural communication, and learning strategies as its main contents, and integrates various teaching modes and methods [9]. It is evident that English language knowledge and intercultural communicative competence are complementary and inseparable, and the challenges and tasks faced by college English teaching are to enhance cultural awareness, enrich cultural knowledge, and cultivate intercultural competence [10]. It can be found that the English curriculum requirements in both the high schools and universities of China attach much importance to the goal of intercultural communication by means of cultural teaching. However, few previous studies explored the characteristics and trends of EFL education from the perspective of cultural teaching.

Co-citation analysis involves examining the relationship of the articles cited by the same articles. The information is explained and analyzed by subject professional knowledge, exploring its development law and predicting its development trend [11]. Co-occurrence analysis involves examining the frequency with which two terms or keywords appear together in a set of documents. Based on the frequency of occurrence of subject terms in a set of literature, a co-occurrence keywords network, which macroscopically reflects the research status and relationships among disciplines and subjects in the field, can be drawn [12]. With the help of the concepts of closeness, intermediary, and centrality of the network, it is possible to analyze important research, authors, and group distribution [13].

This study uses CiteSpace, a visualized analysis tool, to conduct a bibliometric analysis of research literature related to EFL cultural teaching based on the core database of Web of Science (WoS). By analyzing the bibliometric graphs related to EFL cultural teaching in the international scholarship, this study aims to analyze the research distribution, hot topics, and evolution of frontier research in the field of cultural teaching. Therefore, this study focusses on two questions: what are the characteristics of the research on EFL cultural teaching in the international scholarship? And accordingly, what lessons can EFL education learn?

## 2. Literature Review

A number of studies investigated EFL education from technological and psychological perspectives. The application of information technologies was much explored to aid EFL education; for example, video-based virtual reality (SVVR) [14], online teaching [15,16], digital games [17], social media [18], Intelligent Personal Assistants (IPA) like Google assistant [19], corpus software [20], computer-assisted teaching [21,22], gamified flipped classroom [23], Google classroom [24], and authoring systems of robots and IoT-based toys [25]. Foreign language acquisition is influenced by affective factors, which include not only positive emotions but also negative emotions [26,27], so many studies investigated EFL education from the perspective of psychology. For example, teachers’ enthusiasm and students’ enjoyment [28], interaction between classroom anxiety and enjoyment [29], Attention, Relevance, Confidence, and Satisfaction (ARCS) model [30], regulation model of self-efficacy, reflection, and burnout among teachers [31], learners’ willingness to communicate [32,33], relationship between students’ attendance willingness and teachers’ credibility [34], teacher resilience [35,36], teaching enjoyment [37,38,39,40], teachers’ perceptions of critical thinking, and teachers’ immunity [41,42]. Teacher education is also one of the core research perspectives. For example, curriculum innovation in teacher education [43], impact of EFL teacher education on teaching practices [44], metaphors in pre-service EFL teacher education [45], social subject construction in EFL teacher formation programs [46], and multimodal teaching practices for EFL teacher education [47].

Many review articles touched on cultural teaching in the overall analysis of English Language Teaching (ELT) or Foreign Language Teaching (FLT). Meadows (2016) conducted a literature review on the topic of culture teaching in ELT with focus on two questions: How is culture defined? And what does culture teaching look like [48]? He had three observations: the role of teacher shifted from the center of cultural knowledge to the facilitator of knowledge, there is a movement between culture-specific to culture-general orientations, and the scholarship in this field has a growing presence of critical theory which underlies the thinking about culture teaching. Gan and Huang (2017) employed CiteSpace to perform a bibliometric analysis of the domestic studies of China on cross-cultural communication from 1996 to 2016 [49]. They found that the definition of cross-cultural communication competence was the hottest topic and empirical research on cross-cultural sensitivity and intercultural communicative competence had become increasingly important. Qian (2017) conducted a qualitative study on the EFL teachers’ beliefs about culture teaching and their instructional practices [50]. The findings revealed that culture teaching is mainly perceived as presenting factual information, the role of culture in EFL education varies with teachers and students, and the teachers lack knowledge of cultural theory and pedagogical skills. Ye (2019) performed content analysis of the EFL distant education literature and found a research focus on blended learning, flipped classrooms, instructional design, and learner characteristics [51]. Graham et al. (2020) made a systematic review of EFL studies by using the children’s literature to teach, i.e., content, communication, cognition, and culture. They concluded that the children’s literature can be used to teach culture by providing students with opportunities to consider different perspectives, make connections, and understand diverse cultural backgrounds [52].

Bibliometric analysis of the research literature regarding cultural teaching is mainly conducted in the scholarship in China. Zhang and Liu (2020) made a visualized analysis of the intercultural teaching research of the CSSCI journals in China from 2009 to 2018 [53]. The findings revealed that intercultural teaching became the research hotspot in 2011, the intercultural teaching had its focus on language teaching and education, and the most research articles were commentary ones lacking empirical research. Pei (2021) made a bibliometric analysis of the domestic literature of China regarding integration of Chinese culture into FLT from 1992 to 2020 [54]. The observations are that the academic community has not been formed, the research topics and content are still limited, and the research objects are limited to college students. He called for expanding research samples, conducting diachronic research, and promoting integration in all levels of schools. Du and Ji (2021) used CiteSpace to conduct a bibliometric analysis on the literature of intercultural education research in China and came up with generic suggestions to keep the future studies compliant with China’s education policies and values, strengthen discipline construction, pay equal attention to the Chinese context and the global perspective [55]. Xie (2022) analyzed the domestic literature of intercultural competence research on Chinese youth from 2000 to 2020 [56]. The analysis was concluded with similar generic suggestions that the future study should continue to promote localized research, build a more acceptable and open theoretical system, and provide theoretical support and practical guidance for cultivating youth with intercultural competence.

It can be seen that the international scholarship had conducted little research on the literature of EFL cultural teaching. The Chinese scholarship paid much attention to the literature research of cultural teaching or intercultural teaching; whereas they mostly focused on the literature related to teaching research and practices in China, while overlooking international scholarship. Therefore, it is of much significance to conduct a bibliometric analysis of the literature of cultural teaching and expand the literature scope to the international scholarship, especially those from the EFL countries. By doing so, EFL education can draw on the worldwide theoretic and practical insights of cultural teaching to facilitate its sustainable development.

## 3. Data and Method

Knowledge mapping is a visualization that focuses on a specific domain of knowledge, illustrating the development and structural relationships of scientific knowledge. It possesses dual characteristics of both a ‘graph’ and a ‘spectrum’: the former refers to a visualized knowledge graph, and the latter to a serialized knowledge lineage. Knowledge mapping graphs reveal numerous implicit complex relationships such as networks, structures, interactions, intersections, evolution, and derivation among knowledge units or clusters, and foster the generation of new knowledge [57].

### 3.1. Data

**Figure 1:**
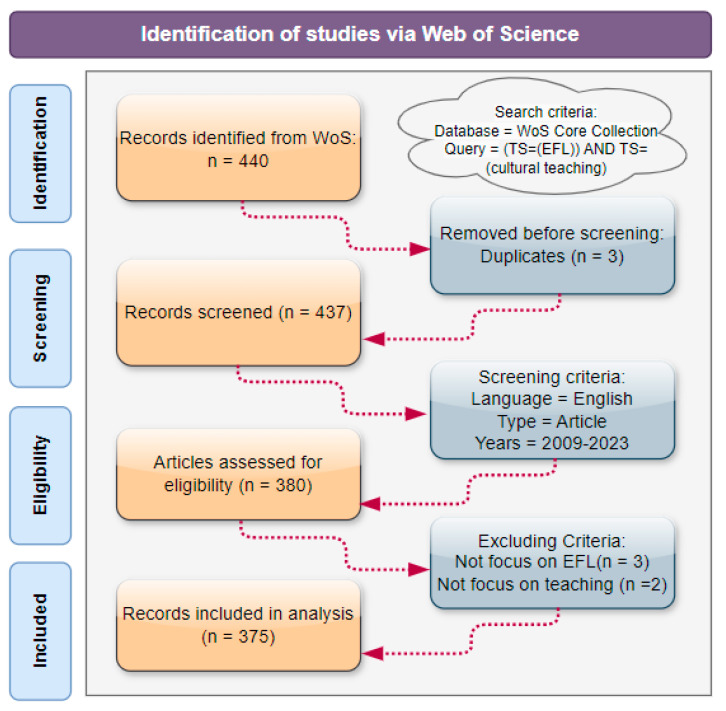
Flowchart of data selection.

Valid data from the literature are the basis for conducting bibliometric mapping analysis. Bradford’s law of literature dispersion states that “most key documents are usually published in a few core journals” [58]. Therefore, this study selects the core collection of Web of Science as the database and the advanced search function is used to retrieve the desired literature data. The search query contains two topics, that is, (TS = (EFL)) AND TS = (cultural teaching). The search results are refined with the following filters, Language = English, Document Types = Articles, Publication Years = 2009–2023. Finally, 375 articles are selected as the data source for analysis. The detailed data selection procedure is illustrated in Figure 1.

### 3.2. Research Method

As a typical knowledge mapping tool, CiteSpace employs multifaceted, time-based, dynamic visualization techniques for citation analysis and pathfinding network algorithms. It not only allows for the representation of the thematic evolution and trend progression of a knowledge domain on a citation network map but also enables automatic identification of key citation nodes in the literature that form the knowledge base, as well as research hotspots characterized by co-citation clustering. In this study, CiteSpace 6.2.R4 was used to conduct a multi-dimensional bibliometric analysis of the objects of countries, institutions, authors, keywords, and co-cited references. The functions of keyword clustering, keyword burst analysis, timeline and time zone maps are used to illustrate research trends, frontier fields, thematic evolution, and hot topics in the field of EFL cultural teaching during the 15 years between 2009 and 2023. This specific period is chosen because the literature before 2009 is too small in quantity which makes it insufficient for trend analysis. In addition to the quantitative research using CiteSpace, the research method of documentation studies is also adopted to analyze the detailed research and practical insights regarding EFL cultural teaching.

**Figure 2:**
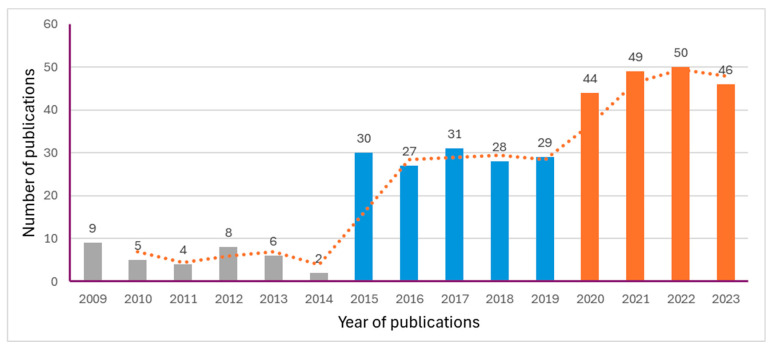
Annual number of publications from 2009 to 2023.

As shown in Figure 2, the annual number of publications has generally shown an upward trend, which indicates a continuously increasing academic interest in EFL cultural teaching. In terms of annual number of publications, the research can be divided into three phases. The first phase is the embryonic period (2009–2014), during which 34 papers were published with an average of 6 papers per year. The second phase is the stable exploration period (2015–2019), during which there was a significant increase in the number of publications, with a total of 145 papers and an average of 29 papers per year. The third phase is the stable growth period (2020–2023), characterized by a larger volume and faster growth of publications, with a total of 172 papers and an average of 43 papers per year. The characteristics of the research on EFL cultural teaching in the international scholarship will be analyzed from three aspects: research distribution, hot topics, and evolution of frontier research.

## 4. Results

### 4.1. Research Distribution

The research distribution of EFL cultural teaching is analyzed with the cooperation networks of regions and authors, and the co-citation network of cited authors.

#### 4.1.1. Research Distribution by Regions

**Figure 3:**
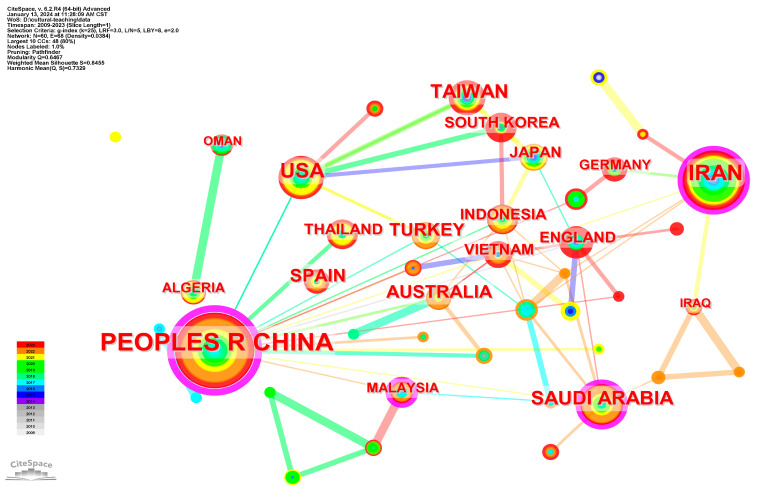
Cooperation network mapping of regions in cultural teaching research.

The selected literature comes from 60 countries or territories. Figure 3 shows the cooperation network mapping of regions, where the weight of spots is determined by the publications of a region, and the weight of links is determined by number of citations. Table 1 lists the regions which have published more than 10 articles, and the centrality of each region. It can be found that China has the largest number of publications and the strongest centrality, both of which are far greater than other regions. It is consistent with the fact that China, as the largest EFL country, attaches the most importance to EFL cultural teaching. The Middle East is another region which is essentially active in the research of EFL cultural teaching, with Iran and Saudi Arabia ranking in top five countries. These two countries have high centrality, which indicates their strong connectivity with other regions. It should be noted that the USA has a large number of publications but low centrality.

#### 4.1.2. Research Distribution by Authors and Co-Cited Authors

The cooperation network mapping of authors can help identify the core researchers and cooperation relationship in a specific research field. On analyzing the cooperation network of authors, it was found that there are two authors who published three articles and the cooperation among authors is loose and scattered. The centrality of all authors is zero, which means that there are no centered authors who contribute to the setup of an academic community in the field of EFL cultural teaching research.

**Figure 4:**
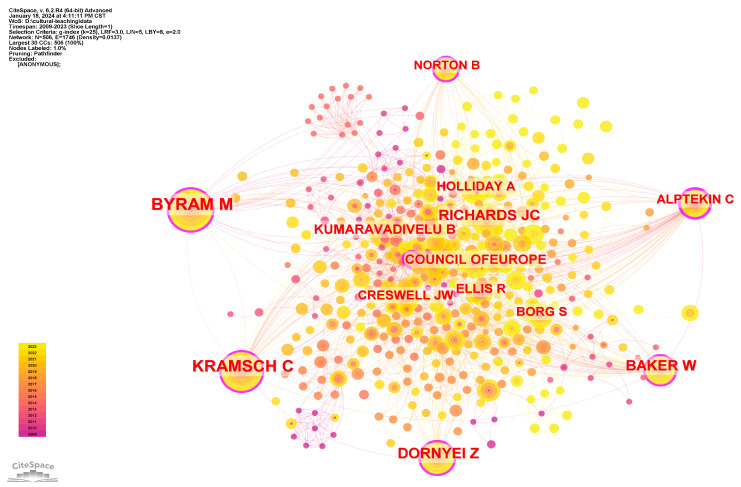
Co-citation network of cited authors in EFL cultural teaching research.

The co-citation network of cited authors can indicate the core researcher in terms of the frequency of being co-cited in different articles. After combing the duplicated cited authors and removing the anonymous authors, we generate the co-citation network mapping of co-cited authors as shown in Figure 4. The top three cited authors are Byram Michael, Kramsch Claire, and Dornyei Zoltan, who has strong centrality as well. Other core cited authors with strong centrality include Baker W., Alptekin C., and Norton B.

Table 2 lists the major cited authors ranked by number of co-citations. There are five cited authors who are co-cited for more than 30 times. It can be seen that all the top cited authors are from English-speaking countries. This means that the significant references from English-speaking countries are far more than those from EFL countries.

### 4.2. Hot Topic Analysis

The hot topic analysis of EFL cultural teaching is conducted with keyword co-occurrence network, keyword co-occurrence clustering, and reference co-citation network.

#### 4.2.1. Keyword Co-Occurrence Analysis

**Figure 5:**
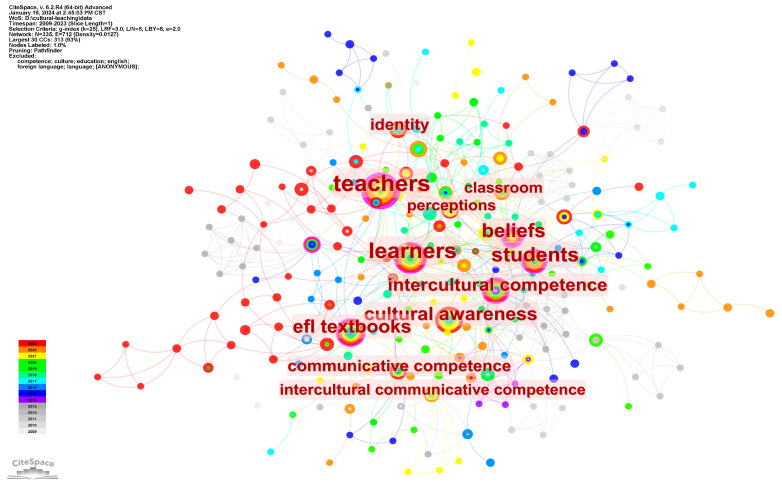
Keyword co-occurrence network in EFL cultural teaching research.

Keywords are the core indicators which reveal the main contents of the articles. The knowledge graph of the keyword co-occurrence network can indicate the hot topics which scholars have studied in a specific field. After excluding the three generic keywords of “language”, “English”, “education”, “culture”, “competence”, “English language teaching”, “EFL”, and “foreign language”, and combining the similar keyword pairs of EFL learners and learners, EFL teachers and teachers, cultural awareness vs. awareness, the CiteSpace generates the keyword co-occurrence networking mapping of EFL cultural teaching research, as shown in Figure 5. A circle represents a keyword, its size represents the occurrences of keywords, and the font size indicates the centrality. The network mapping contains 335 keywords and 712 links with a density of 0.0127. The top keywords of “teachers”, “learners”, “students”, and “EFL textbooks” have strong centrality and can be seen as the participants and resources of cultural teaching or learning. Other top keywords all relate to interculturality, such as “beliefs”, “cultural awareness”, “intercultural competence”, and “identity”.

#### 4.2.2. Keyword Clusters Analysis

**Figure 6:**
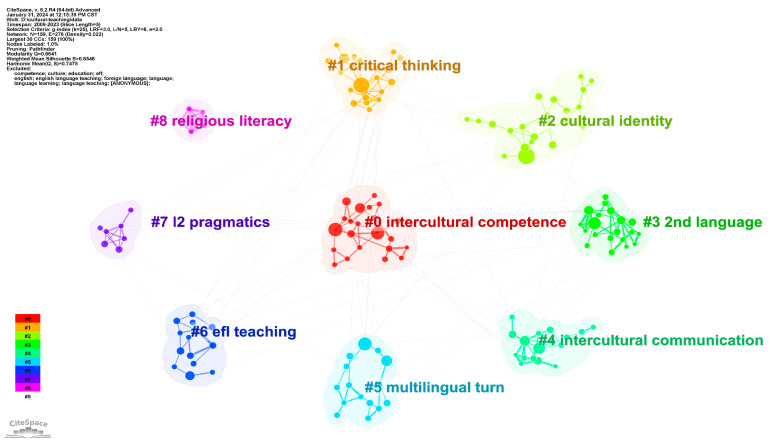
Cluster view of keyword co-occurrence with 5 years per slice.

The keyword clustering map groups and visualizes the related keywords in the form of clusters based on keyword co-occurrence within a set of literature. It can help identify the overarching research topics, infer the relationships between different associated keywords, and analyze the emerging research trends. To better visualize the core topics from an overall perspective, this study uses the slice of 5 years to analyze the keyword clusters. Based on the keyword exclusion and merging settings for the previous keyword co-occurrence network, we set “Years Per Slice” to 5 and regenerate the keyword co-occurrence network. The keyword clustering map is then generated based on the log likelihood ratio (LLR) algorithm as shown in Figure 6. The map has 159 nodes and 276 links with a density of 0.022. The Modularity Q value is 0.6641 (greater than the critical value 0.3), which indicates good clustering effects. The mean silhouette value is 0.8548 (greater than the critical value of 0.5), which indicates reasonable clustering results. Judging from the number of nodes and the inter-connectivity of clusters, Cluster #0 intercultural competence, Cluster #1 critical thinking, and Cluster # 2 cultural identity are the most prominent research themes.

The detailed information of each cluster is shown in Table 3. Clusters #0 and #4 are both associated to interculturality, which can be combined and relabeled as “intercultural communicative competence”. The two clusters contain such core topics as “intercultural competence”, “intercultural communication”, “cultural learning”, “collaborative learning”, and cultural content. Cluster #1 attaches the most importance to critical thinking with emphasis on both EFL learners and EFL teachers. Clusters # 2 and # 6 focus on cultural identity with more attention to “teacher beliefs”, “teacher education”, and “teacher efficacy”. Clusters #3, #5, and #7 are associated to the perspective of sociolinguistics of English, which include the core keywords of “2nd language”, “multilingual turn”, “lingua franca”, “L2 pragmatics”, and “pluriliteracies education”. Cluster #8 is associated with religion, which appears much as a standalone theme in the Islamic world.

### 4.3. Evolution of Frontier Research

**Figure 7:**
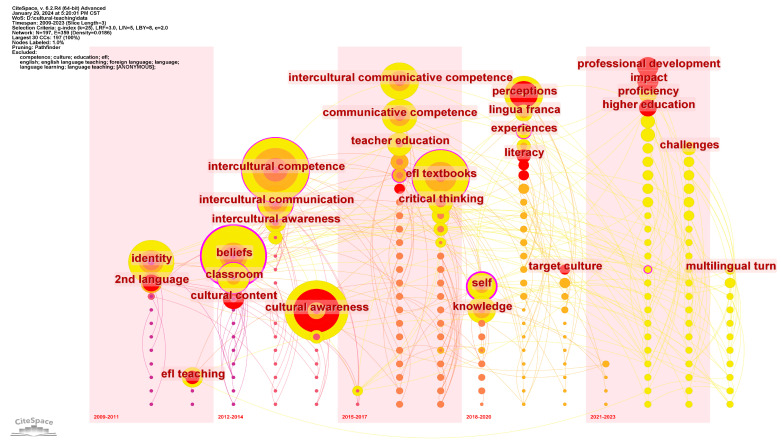
Time zone view of keyword co-occurrence with 3 years per slice.

The time zone view of keywords can visualize the hot topics over time and thus illustrate the evolution of frontier research in different periods. In the 15-year time span, this study takes 3 years per slice to better present the evolution of frontier research. Based on the keyword exclusion and merging settings for the keyword co-occurrence network in Figure 5, we set “Years Per Slice” to 3 and generate the keyword co-occurrence network. We hide the generic keywords of “teachers”, “learners”, and “students” and then click the Timeline icon to visualize the keywords in striped time zones, as shown in Figure 7.

From 2009 to 2011, the identity and beliefs of teachers and learners are the most important topics; meanwhile, classroom and cultural content draw on much research attention. From 2012 to 2016, interculturality is the most important perspective, which covers intercultural competence, cultural awareness, intercultural communicative competence, and intercultural awareness. From 2017 to 2020, EFL textbooks become the largest topic, and the cognitive approach comes to the hottest field including critical thinking, self-recognition, and perceptions. From 2021 to 2023, teachers’ professional development and impact are the burst topics. It is worth noting that source culture starts to be emphasized and multilingual turn comes to the front, which calls for research and practical emphasis on learners’ plurilingual identities.

## 5. Discussions

### 5.1. Establishing an Academic Community of Inclusion and Cultural Diversity

The research distribution by regions and institutions revealed that the scholars and institutions from EFL countries in Asia Pacific (e.g., China) and Middle East (e.g., Iran) are actively exploring EFL cultural teaching based on their own social and cultural contexts, rather than passively absorbing research outcomes from English-speaking countries. In terms of research quantity, EFL countries far surpass native English-speaking countries. However, according to the network mapping of authors, the collaboration among authors appears loose, which means that an academic community in this field has not taken shape. The analysis of co-cited authors shows that all the top 10 authors are from English-speaking countries such as UK and USA. This reveals that the scholars from English-speaking countries take dominant positions in terms of research importance. The studies from EFL countries did not attract much attention from the scholars in this field.

Therefore, in the field of EFL cultural teaching, it is important to facilitate the setup of an academic community of inclusion and cultural diversity. Scholars from EFL countries actively participate and collaborate and contribute more practical and theoretical insights to the community from the perspective of source language and culture. Compared to the previous focus on the research from the English-speaking countries, the community should pay more attention to the research and practices from the EFL countries.

### 5.2. Promoting Interculturality of EFL Education

According to the previous keyword co-occurrence and reference co-citation analysis, the core theme of EFL cultural teaching is interculturality, which includes a set of similar topics such as intercultural competence, intercultural communication, and intercultural awareness. The essence of the intercultural approach is “to help language learners to interact with speakers of other languages on equal terms, and to be aware of their own identities and those of their interlocutors” [59]. According to Vygotsky’s sociocultural theory, the successful implementation of a pedagogy depends on effective interaction between teachers and students, as well as the appropriate teaching resources. Human cognitive development is the result of continuous interaction between individuals and sociocultural objects in the environment. Ref. [60] the learning and cognitive process typically takes place between more experienced individuals (usually teachers) and learners (students). Ref. [61] and Vygotsky believed that it is instruments and their mediating role that connect the two participants and facilitate learners’ development. Ref. [62] teachers and other teaching materials, such as textbooks, serve different mediating functions in the teaching process and jointly contribute to the effectiveness and depth of teaching. The keyword co-occurrence analysis result in Figure 5 also shows that “teachers”, “learners”, “students”, and “EFL textbooks” are the top keywords and have strong centrality. Therefore, to better investigate cultural teaching, it is reasonable to analyze the core theme of interculturality from the perspectives of teachers, learners (students), and teaching materials (i.e., EFL textbooks in this study).

#### 5.2.1. Interculturality of Teachers

As the organizers of the classroom and the instructors of students, EFL teachers must first possess strong intercultural competences, so they should keep learning or participate in education programs to promote their competence. Although EFL teachers and instructors tend to include cultural information in their classes, a number of obstacles prevent them from teaching culture in their English classes. Palmer (2015) studied the cultural conflicts in the EFL classrooms of UAE and suggested that native English-speaking instructors develop intercultural competence by familiarizing themselves with the culture of the UAE through training programs or personal research [63]. Since the USA is the culturally prevailing country, the cultural content in textbooks is often limited and biased towards USA culture. EFL teachers need to be more aware of their own cultural biases and incorporate more diverse cultural content into their teaching [64]. Critical thinking is an underlying ability related to intercultural competence. EFL teachers should keep learning to enhance their professional knowledge and skills in teaching critical thinking. Possible measures are explicit instruction and inquiry-based learning on the basis of the social, cultural, and educational realities of a country.

As the core participant of EFL teaching, teachers always experience negotiation of cultural identity. It is necessary to incorporate cultural negotiation programs into teacher education programs to negotiate their cultural identities, develop new visions for their teaching practices, and address emotional and motivational aspects, ultimately contributing to their cultural identity development. Meihami and Rashidi (2020) proposed a negotiated model of cultural identity development for EFL teachers, by involving them in negotiation sessions, which are about discussing and debating cultural issues related to language teaching [65]. Efeolu (2017) conducted a quantitative study on Turkish EFL teachers and proved the positive relationship between teachers’ cultural intelligence and their professional wellbeing [66]. Schools should provide support and resources for teachers to enhance their cultural intelligence, which can contribute to their professional well-being and job satisfaction. 

Possible teaching techniques are summarized according to the hottest research articles. The incorporation of foreign films into classes can facilitate intercultural learning and help to develop students’ intercultural motivation, attitudes, knowledge and awareness [67]. EFL teachers did not have positive or sufficient experiences with the courses developing Culturally Responsive Teaching (CRT) principles, which include developing cultural knowledge, developing a cultural-related teaching methodology, creating cross-cultural communities, and developing affirmative attitudes toward students’ cultural diversities [68]. Corrective feedback is necessary for EFL teaching. However, EFL teachers seem to misunderstand its impact on students. Students were found to be much more positive about explicit types of corrective feedback than their teachers were, and to experience positive emotions when receiving corrective feedback [69].

#### 5.2.2. Interculturality of Students/Learners

The sociocultural theory proposes that the sociocultural environment is the primary and decisive factor in the development of advanced psychological skills [70]. EFL students/learners tend to have their mindset deeply rooted in their native culture and philosophical values, so they encounter multifaceted identities during English learning. With their growing cultural capital and language capabilities, they often experience a clash of ideological struggles and multilayered identity construction [71]. For example, in the Chinese cultural context, it is necessary to set up interactive contact zones to help construct the identity of Chinese learners in their EFL writing practices as well as in EFL teaching and learning [72].

Critical thinking is an underlying ability related to students’ intercultural competence. Ahn (2015) even regarded cultivation of criticality as the heart of the development of intercultural communicative competence and the shaping of global citizens [73]. To promote EFL students’ intercultural competence, it is necessary to facilitate the cultural shift in their thinking by promoting critical thinking skills, encouraging individual expression and originality, and fostering a student-centered learning paradigm [74].

#### 5.2.3. Interculturality of Textbooks

In the teaching process, textbooks are crucial instruments for imparting knowledge and nurturing talent. Textbooks provide systematic content and frameworks for the entire teaching and learning process, thus becoming a pillar for teachers [75]. They play a key mediating role in cultural inheritance and knowledge transfer. In many countries, it is textbooks that define the “spiritual heritage and legitimate culture” [76]. Textbooks determine what students learn in a large sense because they are seen as authoritative, accurate, and essential references [77]. EFL textbooks serve as the core foundation for cultural teaching, and scholars pay significant attention to whether cultural content in EFL textbooks can help enhance students’ intercultural competence. The interculturality of textbooks implies cultural equality and diversity. Many scholars tried to arouse people’s vigilance about cultural bias. For example, the textbooks in the Headway series have inadequacies and biases when it comes to the presentation and development of intercultural knowledge, attitude, and awareness [78]. The cultural content of some EFL textbooks in Poland does not provide ample opportunities for students to explore and analyze diverse cultures [79]. Cross-cultural understanding receives scant attention in the Top Notch series of textbooks [80]. Soto-Molina and Méndez (2020) analyzed the themed content of the six textbooks used in Columbia and argued that the textbooks favor the dominant culture of English and cannot offer possibilities to embrace interculturality in ELF teaching contexts [81].

The interculturality of EFL textbooks also indicates cultural diversity. Representation of a single culture in EFL textbooks can hinder learners’ ability to communicate effectively in diverse international contexts; therefore, EFL textbooks should offer a more inclusive and diverse perspective to enhance learners’ cultural awareness [82]. In other words, EFL textbooks should incorporate topics and themes that are relevant and relatable to learners from diverse cultural backgrounds. EFL textbooks play an important role in fostering students’ critical thinking, but many of them cannot contribute much to the cultivation of critical thinking.

### 5.3. Highlighting the Multilingual Trend

Multilingual turn is a notable emerging trend in recent years, as demonstrated in Figure 7, the timeline view of keyword co-occurrence clusters. This indicates the scholars’ shifting attention from the English language and culture to the source language and culture, as well as cultural diversity. Al-Obaydi (2019) emphasized the importance of cultural diversity in Iraqi EFL teaching to improve students’ cultural awareness. He suggested to integrate more authentic Iraqi culture content into the curriculum [83]. By doing so, students can be provided with firsthand exposure to their own culture and have a deeper understanding and appreciation of their own culture. CRT is a valid teaching approach compliant with the multilingual and multicultural trend. This approach is to use students’ customs, characteristics, experience, and perspectives as tools for creating learning environments that validate and reflect the diversity and identities of students. However, EFL teachers did not have positive or sufficient experiences with the courses developing CRT principles, including developing cultural knowledge, developing a cultural-related teaching methodology, creating cross-cultural communities, and developing affirmative attitudes toward students’ cultural diversities [84].

This research trend revealed significant implications for EFL education. The previous EFL education has laid much emphasis on the English language and culture, while less is placed on Chinese culture. This poses a sharp contradiction to the intrinsic requirements of cultural teaching, i.e., interculturality and cultural diversity. Therefore, while emphasizing the acquisition of the English language and culture, EFL education should pay equal attention to the learning of world cultures and the cultivation of communicative competence regarding source culture.

## 6. Conclusions and Implications

Higher educational research is on rise in the Chinese context [83,84,85]. By employing the bibliometric analysis method, this study conducted an in-depth analysis of the literature of EFL cultural teaching and led to the following findings: (1) The EFL countries prevail over the native English-speaking countries in terms of publication quantity, but lag far behind the later in terms of research influence. (2) Academic cooperation among authors appears scarce, so the academic community in this field has not taken shape. (3) The core theme is interculturality, which includes a set of similar topics such as intercultural competence, intercultural communication, and intercultural awareness. The research approaches to interculturality can be categorized as teachers, learners/students, and textbooks. (4) According to the evolution of frontier research, multilingual turn is a notable emerging trend in recent years, which calls for cultural diversity and more attention to source languages and cultures.

The literature has enticed researchers to come forward and contribute towards higher education [86,87,88]. To facilitate sustainable EFL education in higher education, this study ends up with the following suggestions for reference: (1) In terms of academic research, scholars in EFL countries should pay more attention to the research and practices conducted by the scholars from other EFL countries, which carry substantial EFL teaching experiences and insights. (2) Teachers should develop intercultural competence, learning how to teach critical thinking and negotiate their own cultural identities. (3) Students should be guided in identity construction and taught critical thinking. (4) EFL textbooks should reflect cultural equity and diversity and contribute to the development of critical thinking.

This study had substantial theoretical and practical implications. Firstly, the traditional cultures of many EFL countries are mostly less known in the world compared to the culture of English-speaking countries, so cultural diversity and identity are two essential considerations for EFL cultural teaching. Secondly, it is necessary to reevaluate the objective of cultural teaching in the context of EFL education. The objective should be to nurture in students a profound sense of identity and self-recognition toward their national culture and an appreciation for excellent world cultures, on the basis of critical and reflective thinking. The cultivation of intercultural communicative competence aims for mutually reinforced interaction between English language/culture and source languages/cultures.

## 7. Limitations

There are two limitations in this study. Firstly, the scope of the literature in this study is limited to English articles only. However, other languages of research articles from EFL countries can probably provide more substantial theoretic and practical insights. Therefore, the bibliometric analysis in future studies can cover the literature published in the source languages of EFL countries. It is of great significance to make a comparative analysis of the differences between the scholarship of EFL countries and the international scholarship and to investigate the reasons for those differences. Secondly, the themes and topics reflected in the knowledge mapping are analyzed from an overall perspective due to the scope settings of this study. A deeper analysis of the topics and the articles therein can possibly bring more illuminating ideas to EFL education.

## Figures and Tables

**Table 1:** Region ranking by number of publications.

Region	Publications	Centrality
PEOPLES R CHINA	61	0.36
IRAN	42	0.11
USA	30	0.07
SAUDI ARABIA	25	0.18
TAIWAN	24	0.00
TURKEY	19	0.01
AUSTRALIA	18	0.05
SPAIN	17	0.00
ENGLAND	11	0.07
INDONESIA	11	0.06
THAILAND	11	0.00
VIETNAM	10	0.04
JAPAN	10	0.02
SOUTH KOREA	10	0.00

**Table 2:** Cited authors ranking by number of co-citations.

Cited Author	Institution	Co-Citations	Centrality
BYRAM, Michael	Durham (UK)	73	0.12
KRAMSCH, Claire	Berkeley (USA)	63	0.12
DORNYEI, Zoltan	Nottingham (UK)	39	0.11
BAKER, Will	Southampton (UK)	37	0.13
RICHARDS, Jack C.	Victoria Univ (New Zealand)	36	0.03
COUNCIL OF EUROPE	EU	25	0.05
KUMARAVADIVELU B	San Jose Univ (USA)	24	0.07
ALPTEKIN, Cem	NY Univ (USA)	23	0.22
HOLLIDAY, Adrian	Canterbury Christ Church Univ (UK)	23	0.05
CRESWELL, John W.	Nebraska–Lincoln (USA)	23	0.01
BORG, Simon	Univ of Leeds (UK)	21	0.08
ELLIS, Rod	Curtin Univ (Australia)	21	0.07
NORTON, Bonny	UBC (Canada)	19	0.13

**Table 3:** Core keywords in each cluster.

Cluster	Nodes	Year	Keywords (LLR Algorithm)
0	22	2015	intercultural competence (14.26, 0.001); cultural learning (7.18, 0.01); collaborative learning (7.18, 0.01); television (7.18, 0.01); chinese efl learners (7.18, 0.01)
1	22	2016	critical thinking (10.92, 0.001); efl learners (7.65, 0.01); efl teacher (7.26, 0.01); adult learning (7.26, 0.01); oral corrective feedback (3.8, 0.1)
2	21	2019	cultural identity (7.96, 0.005); teacher beliefs (7.96, 0.005); pre-service teacher education (7.96, 0.005); saudi arabia (7.96, 0.005); culturally responsive teaching (7.96, 0.005)
3	20	2016	2nd language (8.33, 0.005); challenges (8.33, 0.005); knowledge (4.15, 0.05); teacher value beliefs (4.15, 0.05); behavioral patterns (4.15, 0.05)
4	17	2016	intercultural communication (22.86, 1.0E-4); cultural content (14.05, 0.001); critical discourse analysis (9.33, 0.005); elt textbooks (9.33, 0.005); intercultural awareness (5.72, 0.05)
5	15	2019	multilingual turn (13.24, 0.001); language learning (10.53, 0.005); english as a lingua franca (8.8, 0.005); migrant efl learners (8.8, 0.005); pluriliteracies education (8.8, 0.005)
6	14	2017	efl teaching (11.17, 0.001); teacher efficacy (10.3, 0.005); cross-cultural analysis (10.3, 0.005); impact (5.13, 0.05); individual differences (5.13, 0.05)
7	7	2020	l2 pragmatics (7.51, 0.01); elf (7.51, 0.01); business professionals (7.51, 0.01); belf (7.51, 0.01); metaphoric mapping (7.51, 0.01)
8	5	2016	religious literacy (9.71, 0.005); cultural dimensions (9.71, 0.005); curricular program (9.71, 0.005); observance of religion (9.71, 0.005); value spirituality (9.71, 0.005)

## Data Availability

The original data presented in the study are openly available in Web of Science, which is an open academic database. Section 3.1 Data explained the way to duplicate the research data.
